# Cannabinoids and Hormone Receptor-Positive Breast Cancer Treatment

**DOI:** 10.3390/cancers12030525

**Published:** 2020-02-25

**Authors:** Luka Dobovišek, Fran Krstanović, Simona Borštnar, Nataša Debeljak

**Affiliations:** 1 Institute of Oncology Ljubljana, Zaloška cesta 2, SI-1000 Ljubljana, Slovenia; 2 Medical Centre for Molecular Biology, Institute of Biochemistry, Faculty of Medicine, University of Ljubljana, SI-1000 Ljubljana, Slovenia

**Keywords:** hormone receptor, breast cancer, cannabinoids, treatment, CBD, THC, estrogen, cannabinoid receptor

## Abstract

Breast cancer (BC) is the most common cancer in women worldwide. Approximately 70–80% of BCs express estrogen receptors (ER), which predict the response to endocrine therapy (ET), and are therefore hormone receptor-positive (HR+). Endogenous cannabinoids together with cannabinoid receptor 1 and 2 (CB1, CB2) constitute the basis of the endocannabinoid system. Interactions of cannabinoids with hypothalamic–pituitary–gonadal axis hormones are well documented, and two studies found a positive correlation between peak plasma endogenous cannabinoid anandamide with peak plasma 17β-estradiol, luteinizing hormone and follicle-stimulating hormone levels at ovulation in healthy premenopausal women. Do cannabinoids have an effect on HR+ BC? In this paper we review known and possible interactions between cannabinoids and specific HR+ BC treatments. In preclinical studies, CB1 and CB2 agonists (i.e., anandamide, THC) have been shown to inhibit the proliferation of ER positive BC cell lines. There is less evidence for antitumor cannabinoid action in HR+ BC in animal models and there are no clinical trials exploring the effects of cannabinoids on HR+ BC treatment outcomes. Two studies have shown that tamoxifen and several other selective estrogen receptor modulators (SERM) can act as inverse agonists on CB1 and CB2, an interaction with possible clinical consequences. In addition, cannabinoid action could interact with other commonly used endocrine and targeted therapies used in the treatment of HR+ BC.

## 1. Hormone-Receptor Positive Breast Cancer 

Breast cancer (BC) is the most common cancer in women worldwide [1]. Approximately 70% to 80% BCs express estrogen receptors (ER) and are therefore hormone receptor-positive (HR+). Furthermore, 65% of these cancers are also progesterone receptor (PR)-positive and PR expression is used as a biomarker of ER signaling [2,3]. Expression of ERs predicts the efficacy of endocrine therapy (ET), which is the cornerstone of the management of HR+ BCs [4,5,6]. One third of tumors that express ERs have primary resistance to treatment with ET, and in the long term, most of the patients develop secondary resistance [7]. ERs are steroid receptors that bind various endogenous (17β-estradiol, estrone, estriol, estetrol) and exogenous estrogens or mimetics. Two types of ERs have been identified; ERα and ERβ. BC oncogenesis is mediated primarily by ERα [8]. ERs act as a transcription factor that translocates into the nucleus and binds with estrogen-response elements (ERE). ERα-regulated gene expression promotes cancer cell proliferation and cell viability [9]. The activation of ERβ has antiproliferative effects in hormone receptor-positive MCF-7 and T-47D BC cell lines. ERβ overexpression downregulates cell cycle-related genes and DNA replication. ERβ inhibits cell proliferation by c-myc, cyclin D1, and cyclin A gene transcription repression and causing an increase in expression of p21 and p27, inducing G2 cell cycle arrest [10]. 

## 2. Cannabinoids and the Endocannabinoid System 

Cannabinoid receptors (CBRs) are membrane G-protein coupled receptors (GPCR). Cannabinoid receptor 1 (CB1) was discovered in 1988, which was followed by the discovery of cannabinoid receptor 2 (CB2) in 1993. Recent studies have shown that cannabinoids can activate other receptors, i.e., GPR18, GPR119, TRPV1, and GPR55 which is considered by some as a CB3 receptor [11,12,13]. CBRs can be activated by endogenous or exogenous cannabinoids, which can be of natural or synthetic origin. Endogenous cannabinoids are substances produced by the human body. The most studied are N-arachidonoylethanolamine (anandamide) and 2-arachidonoylglycerol (2-AG). Together with CBRs, the endogenous cannabinoids constitute the basis of the endocannabinoid system [14]. Delta-9-tetrahydrocannabinol (THC) is the main psychoactive component of *Cannabis sativa* and is therefore an exogenous phytocannabinoid and a non-selective agonist of CB1 and CB2 [15]. Cannabidiol (CBD) is another phytocannabinoid abundant in *Cannabis sativa* and is emerging as potential therapeutic agent [16]. In comparison with THC, it displays lower CB1 and CB2 affinity and acts as an inverse agonist at the CB2 [17]. Synthetic cannabinoids are a heterogeneous group of substances and can be selective agonists of CB1 or CB2 [18,19]. Synthetic THC analogue dronabinol is used in palliative treatment (alongside the standard therapy) for hard to manage symptoms of anorexia, weight loss, and sleep disorders [20,21].

## 3. Cannabinoid Receptor 1 

**Figure 1:**
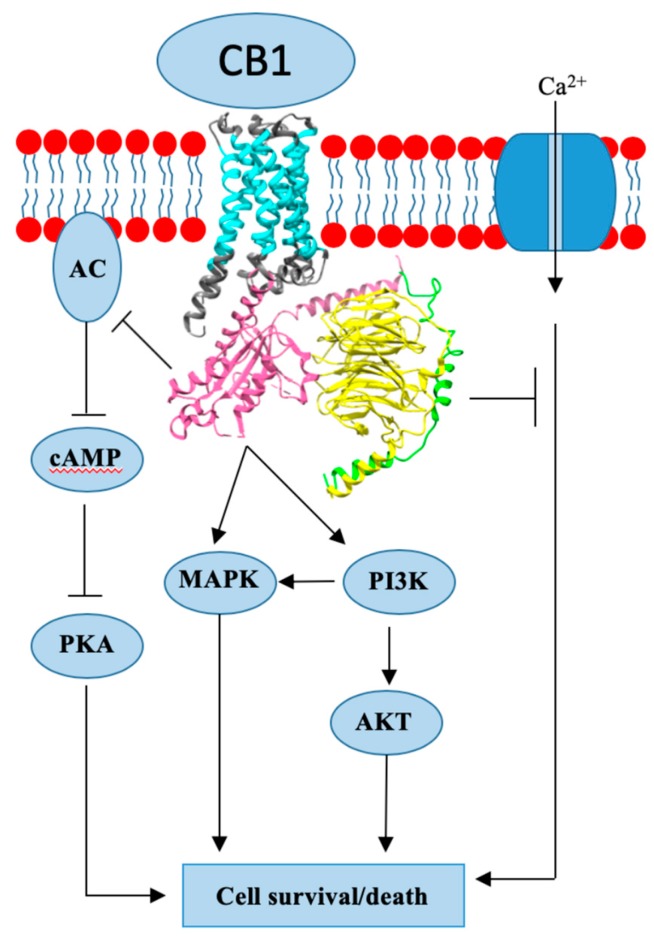
Cannabinoid receptor 1 (CB1) crystal structure and mode of action.

CB1 is a GPCR associated receptor [22]. The receptor is encoded by the gene *CNR1*, which is referred to as a canonical sequence, due to the identification of two other CB1 splice variants [22,23,24]. Canonical CB1 expression and function is best described in the central and peripheral nervous system. CB1 expression is not limited to the nervous system, as expression is present in other peripheral tissues, i.e., cardiovascular, gastrointestinal, immune system, skeletal muscle, pancreatic, fat tissue, etc. The function of CB1 in the majority of the tissues is still under investigation [22]. Apart from widespread localization across the body, the CB1 is shown to have different localization sites on the cellular level. CB1 is dominantly localized on the plasma membrane, but further research has shown that internalized (endosome) and intracellularly (mitochondria, lysosome) located receptors are also present. These subpopulations are shown to have diverse functions from membrane bound CB1. CB1 is a Gi/o type of GPCR (Figure 1), which means that once activated, it inhibits adenylyl cyclase (AC) activity and blocks the accompanying pathway of cyclic adenosine monophosphate (cAMP) formation and protein kinase A (PKA) activation (Figure 1). Another inhibitory function of CB1 is the ability to suppress an influx of Ca2+ ions by closing voltage-gated calcium channels. CB1 mechanism is not limited to inhibiting signal pathways: the receptor is shown to activate several proteins from the mitogen-activated protein kinases (MAPK) family and phosphoinositide-3-kinase/protein kinase B (PI3K/AKT) pathway. CB1 regulates physiological processes such as appetite, learning, memory, pain regulation, energy metabolism, reproductive and cardiovascular system functions. In addition, CB1 is expressed in different tissues under pathological conditions, including cancer. [24]. There is evidence of increased CB1 expression in prostate cancer, pancreatic cancer, colon cancer, hepatocellular carcinoma, non-Hodgkin lymphoma, and astrocytoma [25].

## 4. Cannabinoid Receptor 2

**Figure 2:**
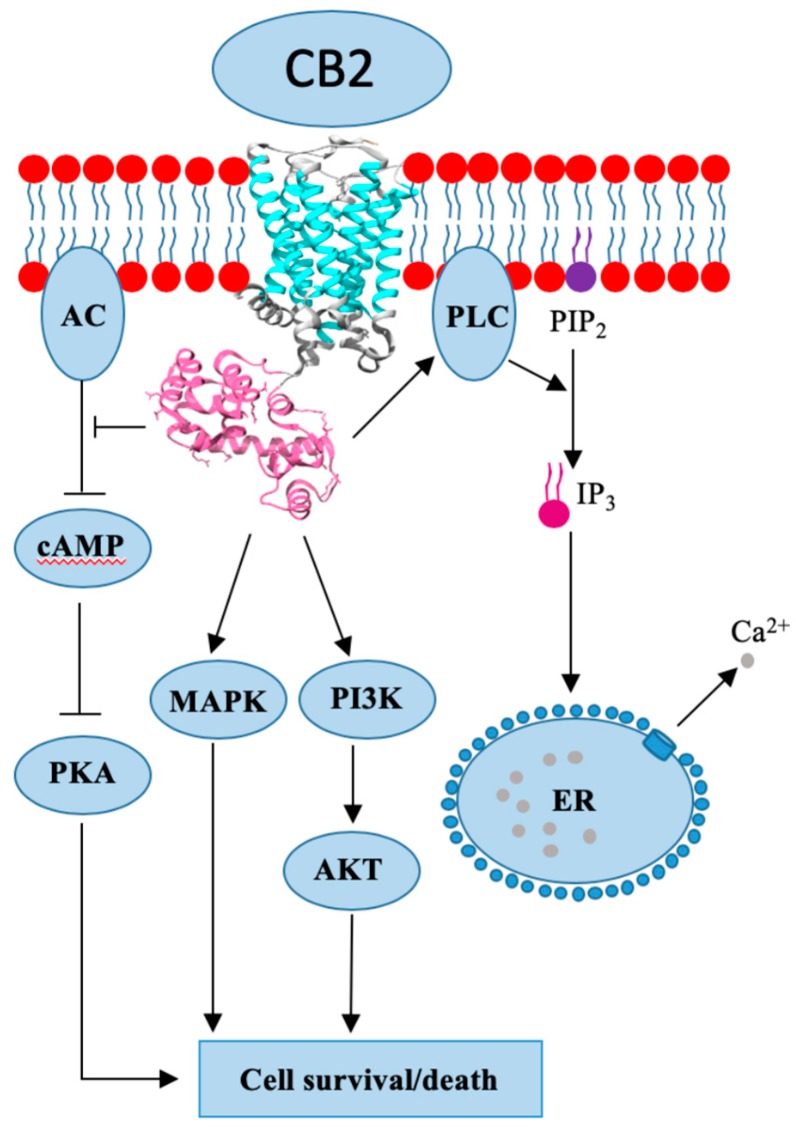
Cannabinoid receptor 2 (CB2) crystal structure and mode of action.

CB2 is a GPCR-associated receptor with two known isoforms and is encoded by the gene *CNR2*. In comparison to *CNR1*, the *CNR2* is shorter and possesses only 44% sequence homology [24]. Isoform CB2A is found in the testis and lower brain regions, while CB2B is more present in tissues of the immune system [26]. Due to its abundance in the immune system, CB2 was discovered in macrophage cells isolated from the spleen [24]. Human leukocytes, such as B- and T-cells, basophiles, eosinophils, mast cells, macrophages, natural killer (NK) cells, and neutrophils have all been shown to express CB2 [24]. Apart from being widely present in the immune system, CB2 can be found in other tissues, i.e., the gastrointestinal tract, cardiovascular and reproductive system, adipose tissue, and in the liver with moderate expression [24]. It was initially believed that CB2 expression is limited to the extracranial tissues, but new research has proven otherwise, as CB2 presence has been found in the brain, although with lower expression intensity. The main function of CB2 is to trigger pro-inflammatory or anti-inflammatory effects in immune cells, depending on the binding ligand, while neural CB2 expression is connected to nociception and neuroinflammation. Even though both CBRs are GPCR (Figure 2), the CB1’s signal pathway is significantly more clarified in comparison to CB2 [23,26]. CB2 was also shown to be a Gi/o type of GPCR, which means that it inhibits AC activity and lowers cAMP levels; however, it is unable to block voltage-gated ion channels (Figure 2). CB2 (just as CB1) is able to activate proteins of the MAPK and PI3K family, and their respective pathways. The receptor is shown to be involved in calcium metabolism by activating the phospholipase C (PLC)/inositol 1,4,5-triphosphate (IP3) pathway, which consequently increases intracellular and mitochondrial Ca2+ levels (Figure 2) [27]. CB2 expression is increased in breast cancer, hepatocellular carcinoma, glioma, and astrocytoma [25].

## 5. Cannabinoids in Connection with the Hypothalamic-Pituitary-Gonadal Axis 

Interactions of cannabinoids with hypothalamic-pituitary-gonadal axis hormones are well documented in animal models. There is evidence that the acute administration of THC lowers serum luteinizing hormone (LH) and gonadotropin-releasing hormone (GnRH) secretion in ovariectomized female and intact male rats [28,29,30]. Lower concentrations of GnRH result in lower circulating estrogen levels. Anandamide produces similar results in both female and male rats [31]. Cannabinoids could modulate the release of GnRH through their effect on hypothalamic GnRH-releasing neurons that have a high density of CB1 and low density of CB2 [32]. Fatty acid amide hydrolase (FAAH) is responsible for anandamide degradation [33] and estrogens decrease FAAH activity in the mouse uterus [34]. Two studies found a positive correlation between peak plasma anandamide with peak plasma 17β-estradiol, LH, and follicle-stimulating hormone (FSH) levels at ovulation in healthy premenopausal women [35,36]. A possible mechanism responsible for this phenomenon is that increased levels of estrogens at ovulation inhibit FAAH activity and consequently increase endocannabinoid plasma levels [37].

## 6. Cannabinoids and Hormone Receptor-Positive Breast Cancer (Preclinical Evidence) 

There is evidence that molecular pathways between CBRs and estrogens overlap, and this could impact pathogenesis of common diseases, including HR+ BC [38]. Most of the preclinical studies have explored the effects of cannabinoids on BC cell lines. De Petrocellis et al. showed that anandamide can inhibit the proliferation of ER positive MCF-7 and T-47D BC cell lines. The anti-proliferative effect of anandamide was due to the inhibition of DNA synthesis and not toxic effects or apoptosis. There was a reduction of cells in the S phase of the cell cycle. Anandamide suppressed prolactin (PRL) receptor synthesis and the prolactin-induced response. The authors concluded that anandamide blocks human BC cell proliferation through the CB1-like receptor-mediated inhibition of prolactin action at the level of PRL [39]. In contrast to these findings, Hanlon et al. found that JWH-015 (CB2 selective agonist) reduced the viability of MCF-7 cells by inducing apoptosis using a calcium-dependent, cell cycle-independent mechanism. In addition, JWH-015 inhibited the MAPK/ERK intracellular pathway [40]. Meck et al. showed that anandamide inhibits AC and activates MAPK in MCF-7 cells, resulting in inhibitory effects on cell proliferation, PRL receptor expression, and tropomyosin receptor kinase (Trk) levels [41]. There is evidence that anandamide and 2-AG inhibit the proliferation of PRL-responsive human BC cells through the downregulation of the PRL receptor [42]. Another study showed that THC fails to activate ERs and reduces 17β-estradiol induced proliferation of the MCF-7 cell line by a probable ER-independent mechanism [43]. THC and CBD are unable to stimulate the EREtkCAT reporter gene transiently transfected into MCF-7 cells and therefore fail to act as agonists at ER [44]. Furthermore, THC inhibits 17β-estradiol/ERα signaling by up-regulating ERβ, and antiproliferative effects on BC may be modulated by expression levels of ERα in the presence of 17β-estradiol. It was suggested that THC could be categorized as a selective ER modulator (SERM) because of its potential to modulate ER interactions [45]. Takeda showed that growth stimulatory effects of THC are mediated by the products of cyclooxygenase 2 (COX-2) and that THC action is modulated by 17β-estradiol. COX-2 and aromatase individually participate in the proliferation of BC cells induced by THC [46]. In most of the studies, non-selective CB1 and CB2 agonists (anandamide, THC) were used and their action resulted in the decreased proliferation of cancer cells. However, Sarnataro et al. showed that rimonabant (a synthetic selective CB1 antagonist) inhibits the proliferation of ER positive BC cells through a lipid raft-mediated mechanism. The growth of the highly invasive metastatic ER negative MDA-MB-231 cell line was more inhibited in comparison to ER positive T47D and MCF-7. The anti-proliferative effect was completely lacking in the absence of the CB1, suggesting that the antiproliferative effect of rimonabant was CB1-dependent [47]. Blasco-Benito et al. evaluated the antitumor efficacy of pure THC with that of a botanical drug preparation made from fresh cannabis flowers. The botanical drug preparation was more potent than pure THC in producing antitumor responses in cell culture and animal models of different BC subtypes, including the HR+ subtype [48]. 

## 7. Cannabinoids and Hormone Receptor-Positive Breast Cancer (Clinical Evidence) 

Perez-Gomez et al. analyzed a large series of human BC tissue sections. CB2 was expressed by 75.6% of human breast adenocarcinomas, regardless of the subtype. CB2 expression was highly associated to human epidermal growth factor 2 (HER2) positive tumors, while no association between CB2 expression and HR+ or triple-negative BC (TNBC) was detected. Interestingly, nontumor breast tissue did not express CB2. In addition, there was an association between the higher expression of CB2 in HER2 positive disease and the decreased overall survival, higher probability of local recurrence and developing distant metastases. This association was not observed in HR+ patients [49]. Andradas et al. found an association between GPR55 expression and basal/TNBC subtype. They analyzed the publicly available The Cancer Genome Atlas (TCGA) microarray data sets and found that women with basal/TNBC and high tumor GPR55 mRNA expression had reduced overall survival and reduced metastasis-free survival in comparisson to those with low GPR55 mRNA levels [50]. There is no clinical evidence evaluating the effect of exogenous or endogenous cananbinoids on treatment outcomes and/or disease prognosis of any BC subtype. 

## 8. Cannabinoids and Specific Hormone Receptor-Positive Breast Cancer Treatments 

The standard ET of HR+ BC consists of ovarian suppression with GnRH agonists, SERM tamoxifen, selective ER degrader (SERD) fulvestrant, and aromatase inhibitors (AIs). Mammalian target of rapamycin (mTOR) inhibitor everolimus, cyclin-dependent kinase inhibitors (CDKi) and PI3K inhibitor alpelisib are approved in combination with ET.

### 8.1. Selective Estrogen Receptor Modulators

**Figure 3:**
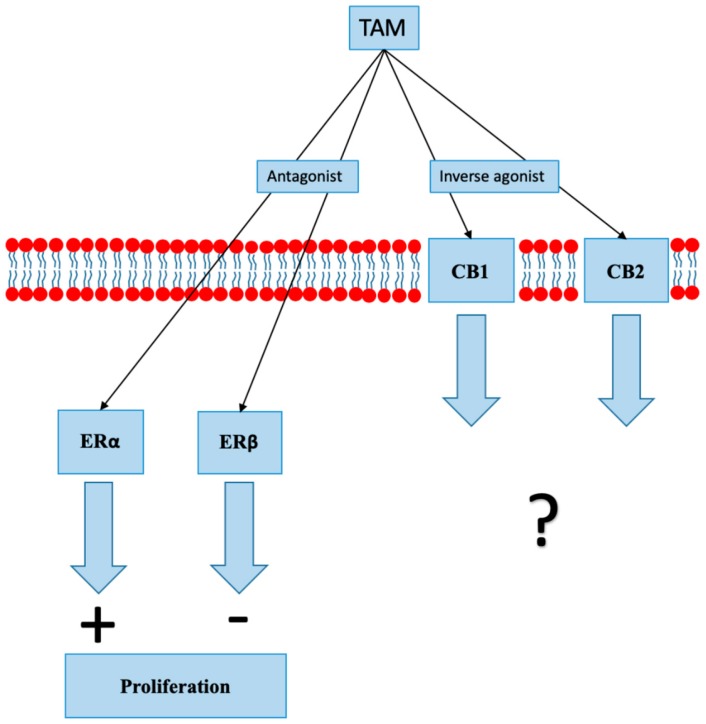
In addition to its action on estrogen receptors (ER), tamoxifen (TAM) acts as an inverse agonist at cannabinoid receptors 1 and 2 (CB1 and CB2). The clinical significance of inverse agonist action on cannabinoid receptors is unknown.

SERMs are synthetic nonsteroidal exogenous compounds that bind to ER with high affinity and block estrogen binding, consequently inhibiting ER-mediated gene expression. Treatment with SERMs, tamoxifen in particular, has decreased mortality due to BC by 25%–30% [51]. Tamoxifen acts as an antagonist at ERα and ERβ [52,53]. Other tamoxifen actions include an increase in cellular oxidative status, inhibition of protein kinase C (PKC), elevation of cytosolic and mitochondrial calcium levels, modulation of mitogen-activated protein kinase 8 (MAPK 8) activity, and induction of transforming growth factor beta (TGF-ß) production and secretion [52]. Many mechanisms associated with resistance to tamoxifen have been identified; they include mutations in genes encoding ERs and changes in signaling pathways that lead to ER independent signaling [54]. Two recent studies have shown that tamoxifen and several other SERMs can act as CB1 and CB2 modulators (Figure 3). Tamoxifen and its metabolite 4-hydroxy-Tam (4-OH-Tam) bind to CB1 and CB2 with a moderately high affinity, reducing AC inhibition produced by constitutively active CBs [55,56]. Raloxifene, which is a SERM used in the prevention of BC, also acts as a CB2 inverse agonist [57]. 

Blasco-Benito et al. applied a combination of THC or cannabis drug preparation with tamoxifen to ER positiveT47D cell cultures. Submaximal concentrations of tamoxifen in combination with pure THC and cannabis drug preparation decreased the viability in an additive manner. The additive effects observed between tamoxifen and cannabinoids in cell cultures was not evident *in vivo* [48]. There are no clinical studies evaluating the effect of cannabinoids on treatment with tamoxifen. 

### 8.2. Gonadotropin-Releasing Hormone Agonists

GnRH agonists are used for ovarian suppression in premenopausal women with BC. They are used in combination with tamoxifen or an AI. GnRH agonist work by decreasing the release of gonadotropins from hypophysis and in consequence inhibiting production of estrogens by the gonads. Acute administration of THC decreases serum LH and GnRH secretion in ovariectomized female and intact male rats [29,30]. Anandamide and 2-AG produces similar results in both female and male rats [31]. After their release, anandamide and 2-AG are transported into GnRH neurons that express CB1 and CB2 and are coupled to Gi/Go proteins. The activation of CBRs in GnRH neurons leads to the inhibition of GnRH secretion. CBR agonist WIN 55,212-2 can block the pulsatile release of GnRH from the immortalized GnRH neurons. When a CB agonist CP 55,940 is delivered into the third ventricle of adult female mice, estrous cycles are prolonged by at least 2 days [32]. 

### 8.3. Aromatase Inhibitors 

AIs lower plasma estrogen concentration through the inhibition of the aromatase, which is an enzyme that converts androgens to estrogens in the peripheral tissues. As estrogens are predominantly produced in peripheral tissues of the body in postmenopausal women, AIs are the standard option in the treatment of postmenopausal women with HR+ BC in all settings [58,59]. Takeda et al. reported the modulation of THC-induced BC cell growth by cyclooxygenase and aromatase in the ER positive MCF-7 BC cell line. 17β-Estradiol produced by aromatase interferes with THC-induced cell growth, which is more prominent in low 17β-estradiol environments. THC-mediated BC cell growth is stimulated by co-treatment with AIs. It has therefore been suggested that THC could act as an exacerbating agent when co-treated with estrogen-lowering drugs [46]. There are no clinical studies evaluating the effect of cannabinoids on treatment with AI. 

### 8.4. Selective Estrogen Receptor Degraders 

**Figure 4:**
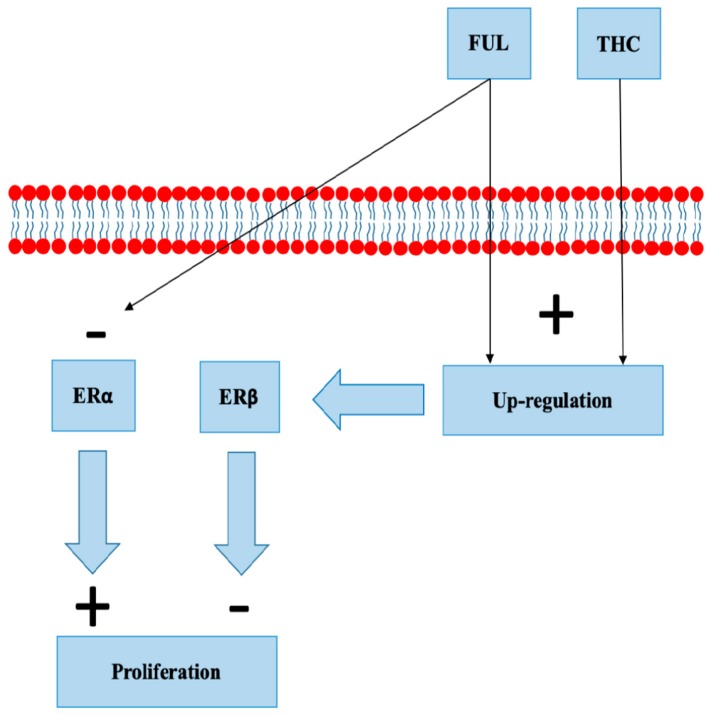
Fulvestrant (FUL) and tetrahydrocannabinol (THC) both up-regulate estrogen receptor beta (ERβ). FUL increases degradation of estrogen receptor alpha (ERα).

Fulvestrant binds to ERα, blocking its dimerization, DNA binding, and nuclear uptake. In addition, it increases ERα degradation with protein degradation processes [60,61]. Fulvestrant is used in the treatment of metastatic HR+ BC in postmenopausal women [62]. Fulvestrant increases ERβ expression in MCF-7 cell lines and animal models [63]. Takeda et al. demonstrated a concentration-dependent up-regulation of ERβ mRNA and protein in MCF-7 cells exposed to THC (Figure 4). Overexpression of ERβ reduced the reporter gene activity of ERα, and its activity was additionally downregulated by THC. The study concluded that THC disrupts estrogen-signaling through the up-regulation of ERβ [45]. There are no clinical studies evaluating the effect of cannabinoids on treatment with fulvestrant.

### 8.5. Inhibitors of Cyclin Dependent Kinases

CDKi are small chemical compounds that inhibit the function of CDKs. CDKs are protein kinases involved in regulating the cell cycle. CDK 4/6 binds with cyclin D to phosphorylate Rb protein. The phosphorylation of Rb protein releases E2F which causes the gene transcription needed for a G1/S transition. Three CDK 4/6 inhibitors (palbociclib, ribociclib, and abemaciclib) are used in the treatment of metastatic HR+, HER2 negative BC. CDK 4/6 inhibitors are indicated only in combination with endocrine therapy (AI or fulvestrant) [64,65,66,67]. Laezza et al. showed that anandamide analogue (Met-F-AEA) induces S-phase cell cycle arrest and decreases the percentage of cells in G2/M phase in the MDA-MB-231 line. This was correlated with checkpoint kinase 1 (CHK1) activation, Cdc25A degradation, and suppression of cyclin-dependent kinase 2 (CDK2) activity [68]. Caffarel et al. showed that THC arrests BC cell lines in G2/M through the downregulation of cyclin-depended kinase 1 (CDK1). In addition, CDK1-overexpressing cells are less sensitive to THC. THC increased the number of cells in the G0-G1 compartment and decreased the number of cells in S phase. Interestingly, the proliferation of normal human mammary epithelial cells was less affected by THC in comparison to BC cell lines [69]. There are no clinical studies evaluating the effect of cannabinoids on treatment with CDK 4/6 inhibitors. 

### 8.6. mTOR and PI3K Inhibitors

PI3K/AKT/ mTOR pathway is the most frequently altered pathway in cancer [70]. Everolimus is an oral protein kinase inhibitor of the mTOR serine/threonine kinase signal transduction pathway and is used in combination with exemestane, a steroidal aromatase inhibitor, for the treatment of HR+, HER2 negative metastatic BC in postmenopausal women [71]. Alpelisib is a selective oral inhibitor of the PI3K catalytic subunit p110α that has shown synergistic antitumor activity with ET against HR+/PIK3CA mutated BC [70] and is used in clinical practice in combination with fulvestrant [72]. Shrivastava et al. found that CBD inhibits AKT and mTOR signaling in TNBC MDA-MB-231 BC cell lines. CBD decreases levels of phosphorylated mTOR, 4EBP1, and cyclin D1 [73]. There is evidence that CBD can suppress the activation of the epidermal growth factor receptor (EGF/EGFR) signaling pathway and its downstream target AKT in TNBC cell lines and animal models [74] and that THC and JWH-133 (selective CB2 agonist) reduce ErbB2-driven BC progression in MMTV-neu mice through AKT pathway inhibition [75]. There are no clinical studies evaluating the effect of cannabinoids on treatment with everolimus or alpelisib.

## 9. Conclusions

Interactions of cannabinoids with hypothalamic-pituitary-gonadal axis hormones are well-documented and two studies found a positive correlation between peak plasma anandamide with peak plasma 17β-estradiol, LH, and FSH levels at ovulation in healthy premenopausal women. There is also increasing evidence that cannabinoids can affect HR+ BC and that ET affects the endocannabinoid system. In most of the preclinical studies, non-selective CB1 and CB2 agonists (i.e., anandamide, THC) were used, which have inhibited proliferation of ER positive BC cell lines. Evidence for antitumor cannabinoid action in HR+ BC in animal models is less clear. Studies have shown that tamoxifen and several other SERMs can act as inverse agonists on CB1 and CB2, an interaction that has possible clinical consequences. There is some clinical evidence indicating CB2 expression in patients with HER2 positive tumors is linked to decreased overall survival, higher probability of local recurrence, and development of distant metastases. Similarly, GPR55 expression in basal/TNBC was linked to reduced overall and metastasis-free survival. Such association was not observed in HR+ BC, however this does not mean that cannabinoids and/or CBRs are not important in HR+ BC setting. Indeed, there are many possible interactions between HR+ BC and exogenous and endogenous cannabinoids. To our knowledge there are no clinical trials evaluating the effect of cannabinoids on BC treatment outcomes and/or prognosis. The interactions between HR+ BC and cannabinoids are complex and the clinical significance of such interactions is currently impossible to predict. Use of cannabinoids in palliative medicine is well established [76], however clinical trials are needed to determine safety of cannabinoid treatment in other BC settings. Until further evidence is available, caution should be exercised by physicians and patients when using cannabinoid preparations in a HR+ (as well as in any other) BC setting.
